# Cross-Species Analysis of Single-Cell Transcriptomic Data

**DOI:** 10.3389/fcell.2019.00175

**Published:** 2019-09-02

**Authors:** Maxwell E. R. Shafer

**Affiliations:** ^1^Biozentrum, University of Basel, Basel, Switzerland; ^2^Department of Molecular and Cellular Biology, Harvard University, Cambridge, MA, United States

**Keywords:** evolutionary cell biology, single-cell RNA sequencing, transcriptome evolution, species comparisons, cell types

## Abstract

The ability to profile hundreds of thousands to millions of single cells using scRNA-sequencing has revolutionized the fields of cell and developmental biology, providing incredible insights into the diversity of forms and functions of cell types across many species. These technologies hold the promise of developing detailed cell type phylogenies which can describe the evolutionary and developmental relationships between cell types across species. This will require sampling of many species and taxa using single-cell transcriptomics, and methods to classify cell type homologies and diversifications. Many tools currently exist for analyzing single cell data and identifying cell types. However, cross-species comparisons are complicated by many biological and technical factors. These factors include batch effects common to deep-sequencing approaches, well known evolutionary relationships between orthologous and paralogous genes, and less well-understood evolutionary forces shaping transcriptome variation between species. In this review, I discuss recent developments in computational methods for the comparison of single-cell-omic data across species. These approaches have the potential to provide invaluable insight into how evolutionary forces act at the level of the cell and will further our understanding of the evolutionary origins of animal and cellular diversity.

## Introduction

Single-cell RNA sequencing has become a powerful and popular tool, yielding rich and informative cell-type atlases of many tissues, and even whole organisms (Cao et al., 2017; Haber et al., 2017; Achim et al., 2018; Zeisel et al., 2018; Sebé-Pedrós et al., 2018b). These experiments have allowed the characterization of hundreds of poorly understood cell types, and identification of previously unknown cellular diversity across multiple species (La Manno et al., 2016; Montoro et al., 2018; Plasschaert et al., 2018). These datasets allow us to ask questions about the origins of cellular diversity, and the evolutionary mechanisms which have shaped cellular form and function. An ultimate goal of these experiments will be to generate cell type phylogenies, describing the evolutionary relationships between cell types (Kin, 2015; Arendt et al., 2019). However, relating information obtained from different sources and different model and non-model organisms is confounded by many technical and biological factors that make comparisons of single-cell data difficult (Marioni and Arendt, 2017; Stuart and Satija, 2019). These include poorly understood forces shaping transcriptome evolution, and complications in assigning orthology and functional conservation between genes across species.

Much of our understanding of cell biology originates from characterizing cells by their functions, gene expression, and lineage relationships (Zeng and Sanes, 2017). Molecular distinctions between cell types, such as protein or gene expression, have become the *de facto* method for categorizing cells, because it is convenient, easily measured, and comparable across models and systems. With recent advances in sequencing, microfluidics, and nano technologies, it is also now possible to profile the transcriptomes of thousands or even millions of cells in a single experiment (Cao et al., 2017; Underwood et al., 2017; Raj et al., 2018; Paolillo et al., 2019). Computational tools have been developed to interrogate these datasets, identifying clusters of cells with similar patterns of gene expression (Andrews and Hemberg, 2018). These clusters are interpreted as distinct cell types, and these methods have done a remarkable job at matching classification systems based on morphology and function (Marioni and Arendt, 2017; Butler et al., 2018; Moussa and Mãndoiu, 2018; Deng et al., 2019).

Though there is debate about whether these transcriptional distinctions are reliable indicators of cellular types or diversity, single cell sequencing technologies are nonetheless very powerful and have the potential to be used to understand evolutionary relationships between cell types across species. Indeed, these technologies have recently been used to compare embryonic brain development in mice and humans, and the evolution of neuronal cell types in reptiles (Pollen et al., 2015, 2019; La Manno et al., 2016; Tosches et al., 2018). Many datasets are also being independently generated from diverse phyla (Achim et al., 2018; Plass et al., 2018; Siebert et al., 2018; Sebé-Pedrós et al., 2018a, b; Ryu et al., 2019).

These diverse datasets necessitate methodologies which can reconcile the technical and biological batch effects inherent in single-cell sequencing technologies. These tools will ideally be able to identify both homologous and divergent cell types between species, and the transcriptional mechanisms involved in their evolution (Marioni and Arendt, 2017). Here, I offer a perspective on the current state of the field of evolutionary cellular transcriptomics, technologies and platforms. This review will specifically focus on computational tools and approaches for combining and comparing single-cell datasets across species.

## Single-Cell Sequencing and Single-Cell Clustering Approaches

Many solutions have been developed for separating, barcoding, and individually labeling cells (Jaitin et al., 2014; Picelli et al., 2014; Soumillon et al., 2014; Svensson et al., 2018). Advances in microfluidic and microwell technologies have offered an incredible increase in throughput, from hundreds of cells to thousands or millions of cells. These technologies involve either encapsulating cells in micro-fluidic droplets, or placing cells individually in microwells, greatly increasing our ability to observe heterogeneity and rare cell types (Islam et al., 2014; Klein et al., 2015; Macosko et al., 2015; Zheng et al., 2017). Techniques such as Sci-RNA-Seq further increase the number of cells analyzed by combinatorically barcoding cells during isolation (Cao et al., 2017). These techniques increase cell breadth at the expense of sequencing depth, which is thought to more reliably identify cellular heterogeneity compared to high-depth sequencing of fewer cells (due to sequencing costs), such as in Smart-seq2 (Picelli et al., 2014).

**FIGURE 1:**
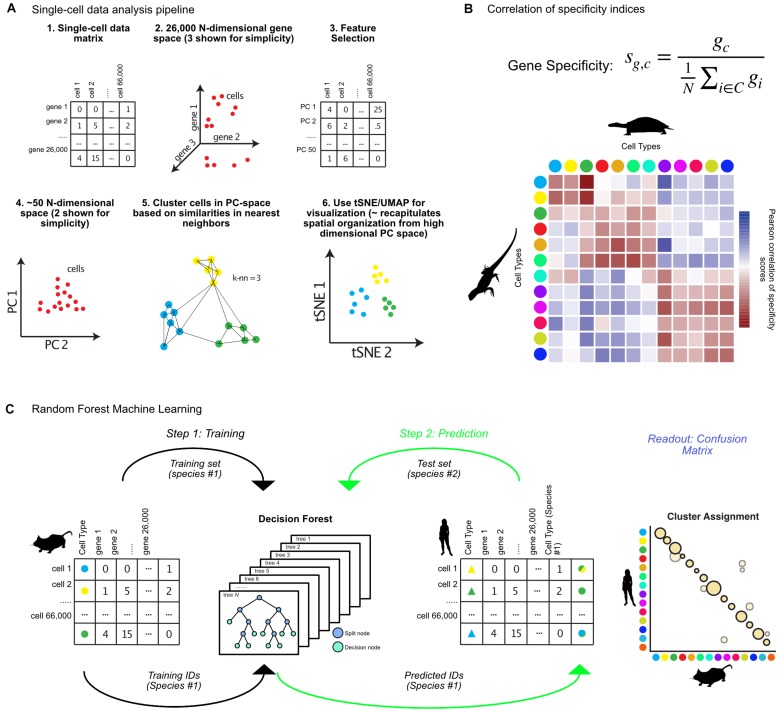
Matching cell clusters in single-cell RNA-seq across species. **(A)** Overview of bioinformatic pipeline for single-cell sequencing analysis from the R toolkit *Seurat*, including feature selection, dimensionality reduction, and graph-based clustering. *Seurat* takes a cell by gene expression matrix (steps 1, 2), and first identifies features (genes) for dimensionality reduction (steps 3, 4). Using principal components, *Seurat* identifies clusters using graph-based methods, then visualizes resulting clusters using tSNE or UMAP (steps 5, 6). **(B)** Equation for calculation of gene specificity, and example correlation of these values between turtle and lizard cell types (colored dots) where Pearson correlation coefficient values in red indicate positive correlation and blue indicate negative correlation. **(C)** Random forest machine learning algorithms for identifying cross-species cell type annotations involves first training an algorithm on cell types from one species (step 1), then predicting which of those cell types each cell from a different species most resembles (step 2), which results in a confusion matrix (Readout). Animal silhouettes were obtained from PhyloPic (www.phylopic.org). All silhouettes were used under the Public Domain Dedication 1.0 license, except the image of a turtle, which is attributed to Scott Hartman.

With the advent of single-cell sequencing experiments numbering in the thousands to millions of cells, sophisticated approaches were needed to deal with statistical challenges in the analysis of the high dimensionality of such datasets. I will briefly describe the main steps taken by the popular single-cell genomics toolkit *Seurat* (Butler et al., 2018). Further information on alternative methods are reviewed elsewhere (Bacher and Kendziorski, 2016; Stuart and Satija, 2019). Many of these packages produce analogous outputs (cluster annotations) which can then be compared across species using the techniques reviewed in the following sections. Initially, the high dimensionality of the datasets are reduced by both limiting the genes under consideration – to so called “highly variable genes,” those which contribute strongly to cell-to-cell variability – and through projection of the data into lower dimensional space using PCA (steps 1–4, Figure 1A; Butler et al., 2018; Yip et al., 2018). The most recent clustering algorithms employ graph-based methods for defining clusters after PCA based on modularity and density of cells within k-nearest neighbor graphs, grouping cells which are mutually close to each other in gene expression space (step 5, Figure 1A; Bacher and Kendziorski, 2016). tSNE or UMAP is used for visualization of clusters, which collapses higher dimensional variability into either 2 or 3 dimensions (step 6, Figure 1A; van der Maaten and Hinton, 2008; Becht et al., 2019).

## Accounting for Experimental and Biological Batch Effects

Comparing and contrasting single-cell datasets will allow for testing the reproducibility of observed biological phenomena, or identification of additional cell type heterogeneity by combining multiple datasets into larger cell-type atlases (Butler et al., 2018; Haghverdi et al., 2018). Comparisons of pharmacological, genetic, and experimental manipulations across different experiments can identify particular and specific gene expression effects and perturbations of cellular states like those observed for disease-associated microglia (Haber et al., 2017; Keren-Shaul et al., 2017; Johnson et al., 2018). Finally, cross-species comparisons of cell types within specific tissues will allow translation of knowledge between model and non-model systems and may suggest evolutionary relationships between cells types both within and between species for the generation of cell-type phylogenies (Marioni and Arendt, 2017).

However, technical batch effects can be introduced at every experimental step, from the cell dissociation procedure, isolation and barcoding, sequencing, and analysis (Bacher and Kendziorski, 2016). In addition to species of origin, biological batch effects caused by differences in genetic background, age, and sex also need to be considered. Several groups have generated computational tools to deal with batch effects specific to single-cell data. These approaches take lessons from the comparison of bulk RNA-sequencing experiments, but have been improved to be able to address the high-heterogeneity of single-cell data (Haghverdi et al., 2018).

## Comparing Cell Types Across Species

**FIGURE 2:**
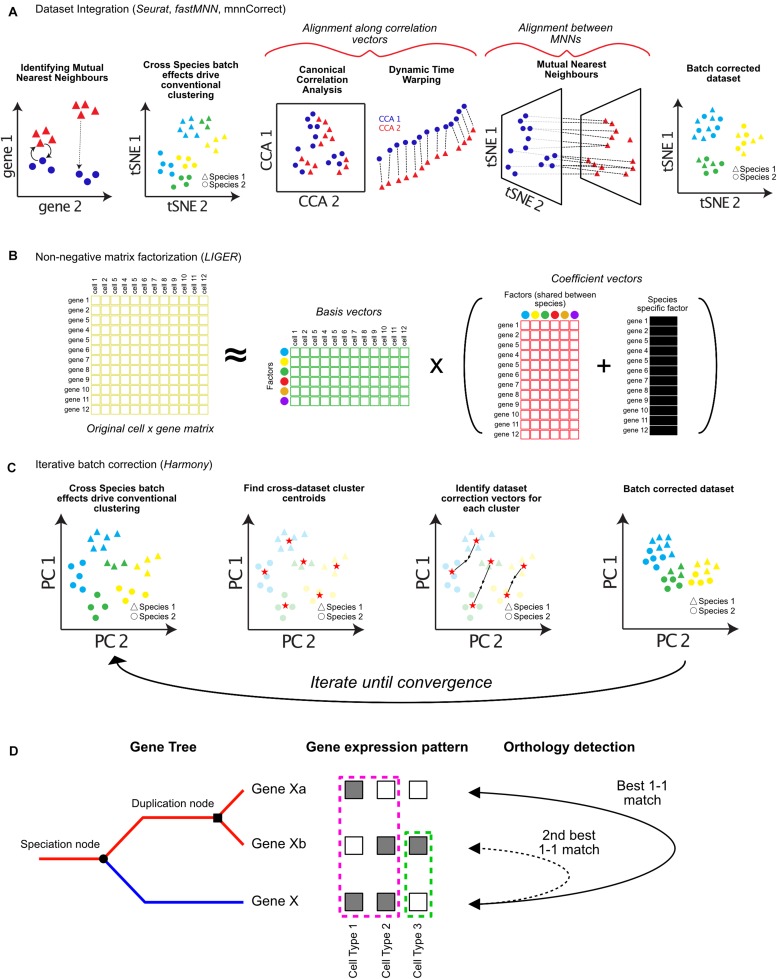
Approaches for integrating single-cell RNA-seq datasets across species. Cells typically cluster by dataset or species of origin, rather than cell types. In order to integrate datasets for downstream analysis, batch correction algorithms can be applied. **(A)** Dataset integration can be accomplished by identifying batch correction vectors using either differences between Mutual Nearest Neighbors (MNN), Canonical Correlation Analysis (CCA), or a combination of both. **(B)** Integrative Non-Negative Matrix Factorization (iNMF) can be used to decompose cell × gene expression matrices into separate factor matrices which can represent species specific factors affecting gene expression patterns. These factors can then be removed to allow clustering by cell types, while retaining information about which genes contribute to species-specific differences. **(C)** Harmony iteratively imputes batch correction vectors based on cell type centroids in Principal Component (PC) space. **(D)** Assigning orthology between genes across species (blue and red lines following speciation node) is complicated by gene duplication events (duplication node). Additionally, sub-functionalization (pink dotted box), or neo-functionalization (green dotted box) of gene expression should be considered when assigning orthology and gene function across species (orthology detection).

Species-specific single-cell datasets can either be analyzed and annotated separately or combined into a single analysis/annotation step. Separate analysis requires cell types to be cross-annotated (typically by hand) but preserves intra-dataset heterogeneity (Figures 1B,C). Combined analyses increase the number of cells used for clustering, allowing identification of additional heterogeneity and rare cell populations. However, it is more complex and computationally intensive, and may obscure species-specific cell types (Figure 2). Combined analyses “batch-correct” the underlying gene expression data, such that the expression levels of genes within cells from each species resemble each other (Haghverdi et al., 2018). In separate analyses, these batch-effects can persist, affecting comparisons and annotations.

In one recent publication, a “gene-specificity index” was used to calculate cross-species pairwise correlation between cell clusters (Tosches et al., 2018). Using a specificity index resolves platform- and species-specific differences in expression quantification, and instead relies on whether a given gene is specific to a cell cluster, or broadly expressed across all cell types (Dunn et al., 2013; Molnar et al., 2013; Kryuchkova-Mostacci and Robinson-Rechavi, 2016). For Tosches et al. (2018) within a set of cell types (*C*), the specificity index (*s*_*g,c*_) of a gene (*g*) for a cell type (*c*) is defined as the ratio between the level of expression of *g* within *c* (*g*_*c*_) and the mean expression of *g* across *C* (Figure 1B). The Pearson-correlation of cell type gene specificity indices can then be calculated, identifying correlated clusters across datasets (red boxes, Figure 1B). The authors used this analysis to compare the pallium, hippocampus, and cortical cell types between turtles, lizards, and mammals. They discovered that mammalian interneuron cell-types were ancestral to all amniotes, but that the mammalian neocortex is largely composed of lineage specific cell types (Tosches et al., 2018).

The previous approach requires cell types to be matched between species by hand, before correlations are calculated. Alternatively, random forest machine learning (RFML) can unbiasedly assign cluster matches across datasets (Breiman, 2001; Denisko and Hoffman, 2018). This has been used to assign cell types across developmental timescales and platforms in the zebrafish habenula, and mouse retina, allowing identification of additional heterogeneity, and differences between larval and adult cell types (Shekhar et al., 2016; Pandey et al., 2018). First, an algorithm is trained to predict the cell types of Species A based on the gene expression matrix generated by single-cell sequencing (step 1, Figure 1C). This produces a set of decision trees, each of which assigns cells to cell types, and which are used to generate a consensus prediction for each cell based on its gene expression signature. This decision forest can then be used to predict the Species A cell types that each of the cells from Species B most resembles. The result of such a comparison is a confusion matrix, which represents the percentage of cells from each cluster in Species B that resemble each cluster from Species A (Figure 1C).

## Computational Integration of Single-Cell Datasets

Even assuming clusters are correctly matched across datasets, comparative analysis of cell transcriptomes remains a difficult task due to batch effects (Stuart and Satija, 2019). Computational integration of datasets allows for unified downstream analysis, however, several factors must be taken into account when removing species-specific batch effects. Most batch correction methods are based on linear regression, which fit a linear model describing the batch effect then impute a new expression matrix without the modeled batch effect (Johnson et al., 2007; Risso et al., 2014; Ritchie et al., 2015). This approach is problematic for single-cell RNA-seq data because it assumes an identical population of cell types within each dataset, and a uniform batch-effect across all cell types (Haghverdi et al., 2018; Welch et al., 2019). Single-cell RNA-seq integration methods must be able to delineate between shared and cell type specific differences between species, and account for differences due to sampling method (number of cells/genes observed, or differences due to dissociation protocols between species). In general, these techniques aim to embed cells from both species into a shared lower-dimensional space, within which clusters and cells can be compared.

The first of such integration methods published, *mnnCorrect*/*fastMNN*, identifies Mutual Nearest Neighbors (MNNs) in high-dimensional gene expression space to identify cell type specific batch-correction vectors (Haghverdi et al., 2018). MNNs are identified as cells which are mutually closest to each other across datasets (Figure 2A). The difference between the expression profiles for each pair of MNN cells is a vector that represents the biological batch effect, and is used to impute new batch-corrected matrices (dotted lines, Figure 2A; Haghverdi et al., 2018).

The R toolkit *Seurat* has also incorporated several methods for dataset integration (Butler et al., 2018). The original *Seurat* alignment procedure involves identifying shared correlation structure across the datasets or species using Canonical Correlation Analysis (CCA) (Figure 2A). CCA identifies groups of genes which have correlated differences in expression. These differences are then used to batch correct each group of genes differently using non-linear dynamic warping, resulting in a shared low-dimensional space (Figure 2A; Berndt and Clifford, 1994). In Seurat v3.0, the authors have incorporated the use of MNNs to aid integration. Following CCA and dynamic time warping, MNNs are identified between datasets and used as “anchors” to compute further correction vectors, similar to *mnnCorrect*/*fastMNN* (Haghverdi et al., 2018; Stuart et al., 2019).

One big issue with these approaches is overfitting during integration, resulting in the merging of cell types, or obscuring dataset-specific gene expression differences. The use of MNNs by both *Seurat* and *mnnCorrect*/*fastMNN* reduces this effect when cell types are present in only a subset of the datasets, because they will not have a mutual nearest neighbor in any other dataset. The panoramic stitching algorithms of *Scanorama* use a more generalized MNN technique, and aim to even further reduce the amount of overfitting between datasets, using a process that is similar to the creation of panoramas from individual images (Hie et al., 2018).

A third method, *LIGER*, uses integrative non-negative matrix factorization (iNMF) to learn shared and unique gene expression signatures between datasets (Welch et al., 2019). iNMF decomposes one matrix (such as a cell by gene expression matrix) into multiple matrices of basis vectors (cell by factor matrix) and coefficient vectors (factor by gene matrix). Factors represent patterns of gene co-regulation, which typically correspond to groups of genes representing specific cell types. For each dataset *LIGER* also infers separate factors that correspond to species-specific signals (Figure 2B). Accounting for species-specific factors allows cell types to be identified across datasets, as well as the characterization of genes which contribute to species-specific differences in each cell type (Figure 2B). In addition to species-specific batch effects, both *Seurat* and *LIGER* can also integrate data across modalities (protein expression, chromatin modifications, and spatial localization) (Stuart and Satija, 2019; Welch et al., 2019).

Finally, several tools have been developed for computationally efficient integration of either extremely large datasets, or an extensive number of datasets. *Harmony* corrects analogous cell types from different datasets toward a shared centroid in low-dimensional PCA space, running iteratively until the datasets converge (Figure 2C; Korsunsky et al., 2018). *Conos* uses a unified graph representation to map cell types across extensive collections of datasets. Spurious connections between datasets are minimized – only cells mapping to each other across multiple datasets are used to identify common subpopulations (Barkas et al., 2018). It will be important in the near future for all of these tools to be benchmarked for different kinds of data, and against each other extensively. I foresee that many of these techniques will be complementary, and that combining approaches will likely be critical for achieving robust performance across many species.

## Incorporating Understanding of Transcriptome Evolution Into Single-Cell Comparisons

Though the above approaches offer exciting possibilities for comparing single-cell data across species, many caveats exist for their implementation. All current approaches require that only the orthologous genes between the species are used during analysis. These genes are used during feature selection and PCA (Figure 1A). Non-homologous genes expressed in only one dataset contribute heavily to variation, and can drive cells to cluster with their own species rather than the same cell type across species (Figure 2C; Stuart and Satija, 2019). However, species-specific information may be lost by excluding genes without one-to-one matches, or with one-to-many matches. Indeed, clade-specific genes are known to drive species-specific cell type diversification (Santos et al., 2017; Florio et al., 2018), and sub- or neo-functionalization in expression patterns of one gene copy following gene duplication is common (Figure 2D; Farrè and Albà, 2010).

For closely related species, such as humans and mice, gene symbols can be easily matched to identify orthologs. For more distantly related organisms, databases such as ENSEMBL can be used to identify one-to-one matches (Zerbino et al., 2018). This works well for closely related species, but becomes more difficult as the amount of evolutionary time between species increases, and the relationship between genes becomes less clear (Thornton and Desalle, 2000). Orthology identification has been largely addressed by the field of phylogenomics – to identify species-relationships and to functionally annotate genomes. Many techniques exist for detection of orthology, most of which are based on sequence-similarity and reciprocal BLAST and other methods reviewed elsewhere (Sonnhammer et al., 2014; Nichio et al., 2017). Incorporating measures of gene orthology or sequence similarity into clustering algorithms will be important to avoid reliance on one-to-one homology for understanding gene function.

Recent work has also identified unique evolutionary forces driving transcriptome variation between species (Liang et al., 2018). Groups of genes with similar regulatory logic are thought to evolve in a modular fashion, with transcriptional changes in these genes linked by the transcription factors which control their expression (Arendt et al., 2016). Some of the integration approaches outlined above may already account for such correlated evolutionarily differences in gene expression (*LIGER*, *Seurat*). Alternatively, removing the most highly correlated genes during clustering analysis may also be a prudent approach (Liang et al., 2018).

## Future Perspectives

The construction of cellular phylogenies should also strive to correctly identify the evolutionary relationships between transcriptionally similar cell types both within and between species. Similarities may result from shared ancestry (homology) or result from convergence onto the same cellular identity (homoplasy). The re-use, re-purposing, or co-option of homologous cellular modules and gene regulatory networks is thought to underlie cell type convergence (Tschopp and Tabin, 2017). Such deep homology not only results in similar cellular functions, but potentially also in highly similar cellular transcriptomes. It may therefore be difficult to disentangle homoplasy from homology using single cell sequencing. Sampling many tissues along larger phylogenies will be necessary to identify where and when specific cell types appear in evolutionary history (Hejnol and Lowe, 2015). From these experiments parsimonious explanations can be developed, providing evidence for homology or homoplasy, and identifying the evolutionary history of specific cellular identities.

Finally, it will be necessary to incorporate phylogenetic comparative methods when comparing differences between species in regard to cell types and gene expression patterns. Biological traits show dependence across species due to the evolutionary history of those species – with more closely related species sharing more similar traits. This should also apply to cell type identities and gene expression patterns (Dunn et al., 2013). Phylogenetic comparative methods account for evolutionary history, modeling trait changes along evolutionary trees, and explicitly take into account their dependence during statistical comparisons (Felsenstein, 2002; Garamszegi, 2014). These have been successfully adapted for bulk transcriptomic data and should be extended to single-cell transcriptomics, where independence of traits is often assumed (Dunn et al., 2013).

## Conclusion

Many techniques, tools, and technologies for single-cell sequencing are already applicable for comparisons across species. However, improvement and refinement of current approaches based on evolutionary knowledge should be considered a priority for the field of transcriptomics and evolutionary cell biology. Understanding the evolutionary history and relationships between cells will provide insight into definitions of cell types, and the molecular mechanisms that govern their identities. Using this evolutionary framework, examining the continuum between developmental stage, cell states, and cell types may even elucidate how cell types evolve (Griffith et al., 2018; Arendt et al., 2019). A holistic identification of cell types and their evolutionary origins will require the combination of multiple lines of evidence, not only including molecular identification, but also functional interrogation, and developmental lineage information. Recent approaches have been developed to reconstruct developmental lineage trajectories *in silico* or using CRISPR barcodes (Briggs et al., 2018; Farrell et al., 2018; Plass et al., 2018; Raj et al., 2018; Wagner et al., 2018; Packer et al., 2019). Incorporating lineage information into evolutionary comparisons will be a difficult, but important task going forward. Such a comprehensive understanding of evolution and cell types will allow us to build cell type phylogenies, and to use them to ask important questions about how cellular changes affect organismal fitness and selection, and how evolution acts on the biological unit of the cell.

## Author Contributions

MS conceived and wrote the manuscript.

## Conflict of Interest Statement

The author declares that the research was conducted in the absence of any commercial or financial relationships that could be construed as a potential conflict of interest.
